# Examining the impact of sex-biased information on health behaviors: a study of HPV vaccination among male college students based on the extended theory of planned behavior

**DOI:** 10.3389/fpubh.2025.1525547

**Published:** 2025-05-09

**Authors:** Tong-Chen Lucas Wang, Mei-Juan Zhang, Hualin Zhang

**Affiliations:** ^1^School of Media and Communication, Shenzhen University, Shenzhen, China; ^2^Institute of Global Communication, Shenzhen University, Shenzhen, China; ^3^Division of Arts, Shenzhen University, Shenzhen, China

**Keywords:** theory of planned behavior (TPB), male HPV vaccination, sex bias, HPV information management, public health campaign

## Abstract

**Introduction:**

Human papillomavirus (HPV) is an exceptionally transmissible virus with a widespread presence that represents a considerable public health concern. Notably, the existing information landscape regarding HPV vaccination tends to favor female perspectives, which may inadvertently neglect the risks associated with HPV infection in men-particularly among male college students, who constitute a vulnerable demographic. To address this gap, effective health communication strategies are essential to encourage vaccination behaviors. This study seeks to extend the Theory of Planned Behavior (TPB) to investigate how sex-biased information influences the HPV vaccination intentions of male college students.

**Methods:**

A survey involving 240 participants was conducted to analyze the correlation between the HPV vaccine information environment and vaccination intentions. Utilizing Structural Equation Modeling (SEM), we assessed both direct and indirect effects that shape this relationship.

**Results:**

Findings reveal that sex-biased HPV vaccination information can have both direct and indirect effects on the vaccination behaviors of male university students. Specifically, attitudes towards the vaccine positively influence the willingness to receive free vaccinations, while subjective norms and perceived behavioral control positively impact the intention to obtain both paid and free vaccinations.

**Discussion:**

This study highlights that the female-oriented focus of HPV vaccination information, which underscores feminine norms, may limit the effectiveness of HPV health education for male college students. Additionally, price considerations have a limiting impact on the favorable attitudes of male university students towards vaccination. Conversely, male-targeted informational campaigns that heighten perceptions of HPV-related risks can diminish price sensitivity regarding vaccines, ultimately fostering increased willingness to vaccinate. The paper concludes by suggesting avenues for future research aimed at developing health communication strategies to enhance HPV vaccination rates among men. Implications and limitations are discussed.

## 1 Introduction

Human Papillomavirus (HPV) infection, primarily transmitted through sexual contact, mother-to-child transmission, and contact with skin and mucous membranes, represents a highly infectious category of viruses. This infection impacts individuals regardless of gender and is associated with various diseases, thereby continuing to present a substantial public health challenge worldwide (1). The epidemiology of HPV in females has been well studied and documented. In contrast, the epidemiological landscape of HPV in males remains less understood. A systematic meta-analysis published in *The Lancet Global Health* underscores the significant prevalence of HPV among the male population. As of 2023, it is estimated that approximately one-third of males worldwide have been infected with at least one variant of genital HPV (2). Furthermore, around one-fifth of males are reported to be infected with one or more high-risk HPV types associated with cancer (2). Cervical cancer, as emphasized by Okunade et al. (3), is one of the malignancies induced by HPV, alongside anal cancer (4) and oral squamous cell carcinoma (5). These HPV-related cancers impose a substantial burden on both the physical and mental well-being of affected individuals. Research indicates that cervical cancer screening data in China reveals an overall HPV infection prevalence rate of 19.1% (6). This prevalence presents a considerable challenge to China’s public health infrastructure, particularly considering its large population, and represents a critical issue regarding the reproductive health of its citizens. In response, China has implemented comprehensive strategies for HPV prevention, which include the National Health Commission’s promotion of free HPV vaccinations for eligible girls, as well as the dissemination of health education and awareness campaigns about HPV through various mediums, notably social media. Nonetheless, the focus of these health initiatives and vaccination campaigns has predominantly been on the female demographic, with insufficient attention directed towards males, who are comparatively disadvantaged in terms of access to vaccine information.

Several studies have shown that men serve as a primary transmission group for HPV (7) and exhibit higher rates of infection (7–10). However, vaccination rates among men remain notably low (11–13). China aims to enhance HPV vaccination efforts among males, and numerous scholars have underscored the importance of this initiative, especially for specific populations such as men who have sex with men (MSM) (11, 13, 14). However, existing literature does not provide clear insights into current vaccination trends and the determinants influencing vaccination among male populations, which constitutes the primary focus of this study.

The observed phenomenon of a high rate of HPV infection coupled with a low vaccination rate among males can be attributed to three primary factors. First, at the individual level, men are less likely to encounter relevant information regarding HPV vaccines within the current information ecosystem, particularly on algorithm-driven social media platforms. Here, discussions surrounding HPV vaccination are frequently linked to ‘cervical cancer,’ a condition specific to the female reproductive system. Even when men do encounter information on these platforms, it is often tailored to a female audience and fails to directly address male health concerns (15). Consequently, the prevailing health information environment emphasizes content and strategies that primarily influence female behavioral intentions, neglecting to engage men as key targets for health promotion. This oversight exacerbates the existing information gap regarding HPV vaccination among males and further diminishes awareness of the implications of sex-biased health information.

Second, at the societal level, the stigma surrounding HPV infection is deeply rooted in prevailing social norms. In the Chinese context, sexually transmitted diseases are often viewed as indicators of a lack of self-respect and self-care, with traditional beliefs disproportionately attributing the responsibility for the occurrence and transmission of these diseases to women (16–18). As a result, women in China are more likely to conform to societal expectations by engaging in preventive measures against sexually transmitted infections, such as HPV vaccination, which has gained acceptance as a mainstream health intervention in recent years. In contrast, societal norms do not similarly impose responsibilities on men, leading to a lower uptake of preventive behaviors among this demographic, including HPV vaccination (112, 113). This dynamic not only reinforces gender norms and stereotypes within sexual health discourse, but also overlooks the significant sexual health risks faced by China’s male population, which numbers approximately 720 million^1^.

Third, from a public health perspective, existing HPV-related health policies in China largely disregard the male population, resulting in a notable gap in media coverage related to men’s health issues. Furthermore, the current public health framework provides various policy incentives for women to receive HPV vaccinations, including free vaccination options for eligible women. In recent years, Chinese public health authorities have been proactively advocating for policies aimed at encouraging men to receive HPV vaccinations. The topic of HPV vaccine pricing for men in China has persistently garnered significant attention and discourse, especially within the dichotomy of out-of-pocket expenses versus free vaccination options. This study also explores the impact of vaccine pricing on the vaccination intentions of males, serving as a preliminary investigation to inform future policy considerations aimed at expanding HPV vaccination access to men.

Considering the previously identified issues, this research enhances the Theory of Planned Behavior (TPB) by integrating sex bias as a precursor variable, while also broadening the scope of HPV vaccination intentions to encompass both self-funded and free vaccination options. This study investigates the mechanism through which sex-biased information affects men’s intentions regarding self-paying for or receiving free HPV vaccinations. The findings aim to offer valuable insights for the future utilization and promotion of HPV vaccines among male populations.

## 2 Literature review

### 2.1 Gendered narratives and systemic gaps in HPV research

HPV vaccination research exhibits a female bias, with a scarcity of male study participants. In studies with a female-oriented focus, scholars have identified social media as a primary source of health information, highlighting that individual engagement with social media content related to vaccines can enhance their awareness of HPV vaccines. A significant positive correlation has been established between the cognitive levels regarding HPV vaccines and the willingness to receive the vaccine among both female populations and parents of young girls (19–21). Additionally, research indicates that vaccination attitudes can influence vaccination behaviors, with parents, as the primary decision-makers regarding their daughters’ HPV vaccination, often hesitant due to uncertainties about vaccine safety (22, 23). Among young women, skepticism regarding the side effects of HPV vaccines emerges as the strongest predictor of vaccination behavior, followed by attitudes towards the vaccination process itself (24, 25). Overall, prior research has consistently demonstrated that the degree of awareness and the prevailing attitudes towards HPV vaccines significantly impact the likelihood of vaccination among female populations.

Vaccination necessitates that individuals engage in rational evaluations of their personal capabilities and the surrounding environmental factors, including economic conditions and the availability of vaccines. Research suggests that the uptake of HPV vaccination among female populations is affected by factors associated with perceived behavioral control. This concept has been shown to be a positive predictor of vaccination behavior among female college students, as well as the vaccination decisions made by parents for their daughters (26, 27). Moreover, self-efficacy, which is closely linked to perceived behavioral control, also plays a crucial role in influencing individuals’ willingness to be vaccinated (28). Environmental challenges, such as shortages of vaccines, pose significant barriers to HPV vaccination, particularly impacting women from low- and middle-income backgrounds (15, 29, 30). Thus, the administration of the HPV vaccine represents a logical decision-making process for individuals, as women consistently assess the effectiveness of their vaccination.

The promotion and administration of HPV vaccines are also shaped by a variety of social and cultural norms, which can exert both positive and negative influences, particularly within Asian societies characterized by intricate family and ethnic dynamics and stringent expectations regarding women’s behavior. Young women from diverse ethnic backgrounds often harbor stigmatizing perceptions of HPV vaccination, including the concern that receiving the vaccine may be construed as an endorsement of promiscuity (31–33). In several Asian nations, discussions surrounding sexual health are frequently suppressed due to prevailing social norms, leading to the perception that HPV vaccination may inadvertently endorse excessive sexual behavior or encourage risky sexual practices (34). Nevertheless, in contemporary Chinese society, certain non-stigmatizing social norms significantly contribute to the encouragement of women’s acceptance of HPV vaccination. For example, within a child-centric cultural framework, Chinese parents demonstrate a willingness to embrace scientific approaches in managing their children’s health, with the moral imperative of ‘scientific parenting’ emerging as a significant predictor of their decisions regarding HPV vaccination (35). Furthermore, health advocacy for HPV vaccination on Chinese social media predominantly targets women (36, 37), while the “feminization” of HPV creates societal expectations that encourage Chinese women to pursue vaccination (38). However, in practice, males, who are notable carriers of sexually transmitted infections, may neglect their own HPV prevention efforts. This negligence can be attributed to the lack of traditional social norms and insufficient media attention regarding HPV vaccination for men. Consequently, they may emerge as new focal points for HPV transmission. This issue represents a significant aspect that has been overlooked in prior research, particularly in studies on sexually transmitted infections conducted in Asia.

The uptake of HPV vaccination is generally shaped by individual factors, particularly among females. These factors include the levels of knowledge, educational background, attitudes towards the vaccine, and personal capabilities. Additionally, social influences, such as cultural norms and the availability of the vaccine, play a significant role. Nonetheless, most existing research has predominantly emphasized the female viewpoint, thereby neglecting male perspectives. This oversight has resulted in a notable gender bias within academic discourse. Consequently, there has been a relative lack of scholarly attention directed towards understanding the disparities in HPV vaccination rates within a sex-biased informational context, particularly regarding the perceptions and health information needs of male populations.

### 2.2 Sex-biased health information environment

Sex bias is characterized by the unequal representation of information and perspectives between genders, typically favoring one gender over the other (39). Sex bias in HPV vaccination information is defined by the unequal distribution of vaccine-related content across male and female populations, whereby the health needs of one gender are prioritized at the expense of the other. Recent studies focusing on the feminization of HPV vaccination (15, 40, 41) have identified three key dimensions in which female-biased HPV vaccination information manifests. Firstly, in the context of information presentation, there is a pronounced emphasis on the significance of vaccination for women, while the infection risks, disease implications, and vaccination advantages pertinent to men are either minimized or overlooked. Secondly, regarding the targeted populations, promotional initiatives predominantly emphasize the protection of women’s health, particularly in relation to the prevention of cervical cancer. In contrast, there is a noticeable lack of attention given to health issues that are relevant to men, such as the prevention of anal cancer, oropharyngeal cancer, and genital warts. Lastly, in framing responsibility for vaccination, there is a tendency to characterize it as a “female obligation,” rather than acknowledging it as a collective public health responsibility that necessitates engagement from individuals of all genders. These three dimensions underscore the notable disparities present in messaging that favors female perspectives over male considerations in HPV vaccination discourse.

Researchers contend that the information landscape of social media—including the dissemination of scientific knowledge, online public discourse, and digital imagery—demonstrates significant sex bias (42–47). Existing studies on sex bias primarily concentrate on descriptive analyses and the identification of influencing factors, aiming to clarify the underlying mechanisms that manifest societal forms of sex bias. Nonetheless, the academic discourse has largely overlooked the implications of sex bias for the promotion of individual health behaviors through information dissemination. The aforementioned study utilizes a feminist perspective to analyze the impacts of male-oriented information environments on women situated within patriarchal structures.

A sex-biased information environment can significantly influence individuals’ attitudes and behaviors, thereby shaping their perceptions of gender role differences. Gender-biased information environments can significantly shape behavioral attitudes by reinforcing existing stereotypes and promoting the internalization of specific beliefs. For instance, the pervasive presence of gender-biased information can influence individuals’ evaluations of certain behaviors through the repeated exposure to concepts such as gender discrimination and stereotypical divisions of labor (48). When these biased messages gain widespread acceptance within society, individuals tend to assimilate them into their personal belief systems. Research supporting this assertion demonstrates that the utilization of sex-biased language plays a significant role in the continuation of gender discrimination and influences individuals’ perceptions and attitudes regarding gender-related concepts (49–51). Furthermore, sex bias is associated with the health outcomes of both men and women (52). In the realm of vaccination, most textual information environments are free from sex bias; for instance, communications regarding flu and hepatitis B vaccines generally concentrate on the diseases themselves rather than on gender distinctions. However, when the information environment pertaining to vaccination decisions is characterized by sex bias, as observed with HPV vaccines, this bias—whether manifested through gender stereotyping or stigmatization—can influence attitudes towards vaccination, thereby impacting both intentions and actual vaccination behaviors. Currently, the information environment surrounding HPV vaccination in China frequently links HPV to female identity, which may lead the public to perceive a greater societal expectation for women, as opposed to men, to receive the HPV vaccine. Social media platforms generate a substantial volume of information daily, varying in quality, which can contribute to a pronounced sex-biased information environment.

The gender-biased information environment influences the subjective norms regarding HPV vaccination among early-adult male college students through gender norms. Firstly, gender roles can be considered a form of social norm (114). Secondly, collectivist cultures emphasize group harmony and adherence to social norms, resulting in individual behaviors being significantly influenced by group opinions (115). Male college students, being in the early stages of adulthood, are particularly sensitive to the perceptions of their peers and experience a strong sense of social identity (116). Within a collectivist context, these male students may be more attuned to socially constructed norms shaped by gender roles and may fear being labeled as non-conforming to these roles. In a gender-biased social media information environment, where HPV vaccination is predominantly viewed as a concern for women, male students may perceive the act of receiving the HPV vaccine as inconsistent with traditional masculine roles, potentially leading to ridicule or social exclusion. As a result, they might avoid vaccination despite being aware of its health benefits. A study investigating the willingness to vaccinate against HPV among Chinese college students studying in the United States revealed that participants more strongly influenced by collectivist culture were less likely to receive the HPV vaccine (53). Therefore, it can be posited that information environments characterized by gender bias have a substantial impact on individuals’ attitudes and subjective norms, especially among individuals embedded in collectivist cultures.

Behavioral attitudes and subjective norms pertain to an individual’s assessment of subjective elements, whereas perceived behavioral control encompasses a consideration of objective factors (54). For individuals, sex-biased information represents an objective reality. The information environment characterized by gender bias can initially diminish individual self-efficacy by influencing perceptions of ability. For instance, the sustained emphasis on male advancement in the fields of science and technology may cause women to undervalue their own competencies within these domains, fostering a belief that “I am not proficient in science and engineering” (55). Furthermore, when the information environment implies that certain resources are preferentially allocated to one gender, individuals may perceive a constricted ability to exercise control over their actions. Specifically, women might show reluctance to pursue executive roles due to prevalent discussions surrounding the “glass ceiling” phenomenon (56). Specifically, information favoring males regarding HPV vaccination and information favoring females influence men’s cognition, particularly among male college students, thereby impacting their perceived behavioral control. Consequently, this study centers on the relationships between sex-biased informational contexts and various factors, including attitudes, subjective norms, perceived behavioral control, and actual behaviors.

Gender roles represent the socialized outcomes of individual behaviors, which are shaped by biological inheritance and influenced by various political, economic, and cultural factors within society. These roles contribute to societal perceptions of gender, with socially prescribed or implicit gender roles guiding individuals’ behavioral patterns through mechanisms such as information dissemination (57). Informed by TPB, some researchers have sought to incorporate gender as a new variable to enhance the TPB framework, investigating its effect on behavioral development within this model. For instance, Kyrrestad et al. (58) integrated gender differences in TPB were detected, Intention to increase the frequency of drinking is predicted by subjective norms (SN) and attitude for girls and by subjective norms (SN) for boys. Similarly, Xu et al. (59) analyzed the differences in learning behaviors between male and female engineering students under peer pressure, and found that gender differences have a positive impact on subjective norms, and high-performing female students can motivate male classmates and improve their learning intentions and behaviors. Under the guidance of TPB theory, Xie et al. (60) tried to explore the influence of gender differences on healthy office building strategies, and the experimental results showed that there was a positive relationship between female employees’ health behavior intentions and architectural design strategies.

These studies primarily focus on gender as a precursor variable within the TPB framework, highlighting its significant impact on individual behavioral patterns. Nonetheless, the mechanisms through which sex-biased information contributes to behavioral differences warrant further investigation. Research suggests that gender can function as a precursor variable influencing individual attitudes, social norms, and perceived behavioral control in the initial stage, ultimately shaping behavioral variations. Furthermore, sex-biased information also impacts societal perceptions of gender, which in turn reinforces attitudes, social norms, and perceived behavioral control, culminating in differentiated behavioral patterns. This study aims to examine whether sex-biased information plays a pivotal role in shaping individuals’ behavioral patterns, with a particular focus on HPV vaccination behavior. This exploration seeks to understand how cognitive differences may lead to varied behavioral orientations.

### 2.3 Extended theory of planned behavior and HPV vaccination

The Theory of Planned Behavior (TPB) is a framework within social psychology that seeks to explain and forecast individual behavioral actions. According to this theory, individuals make decisions regarding their behavior in a rational manner, where three key components—Attitude toward the Behavior (AB), Subjective Norms (SN), and Perceived Behavioral Control (PBC)—interact to shape behavioral intentions. These intentions subsequently influence the likelihood of actual behavior occurring (54). As a well-established theoretical model, the TPB has been widely applied in health communication research, particularly in studies related to HPV vaccination in recent years. Researches have made various modifications and extensions to the theory to better suit different research contexts. Notably, the TPB has demonstrated strong predictive capabilities, providing valuable insights into the processes underlying the formation of new behaviors in specific scenarios, including detailed investigations of HPV vaccination behaviors (27, 61–67).

In the context of deciding whether to receive the HPV vaccination, individuals are required to engage in rational decision-making processes. This involves evaluating their personal health status, considering the economic costs associated with vaccination, and conducting a comprehensive analysis of the potential health benefits. This rational analysis ultimately informs their decision-making process regarding vaccination. Notably, the cost of vaccines has emerged as a significant factor contributing to vaccine hesitancy within the Chinese population (68, 69). In mainland China, HPV vaccines are classified into two categories: free and self-funded. The free vaccines are primarily distributed through the national vaccination program and are currently accessible in 15 provinces. In contrast, the self-funded vaccines are predominantly imported, with an average price exceeding $160, a substantial financial burden relative to the average income of residents in China. Research indicates that the willingness of Chinese women to receive the HPV vaccine is significantly influenced by the cost of the vaccine, with higher prices negatively impacting vaccination rates (70–72). This study specifically aims to investigate the relationship between the willingness to receive the HPV vaccine and its price among males, with the objective of enhancing the understanding of the decision-making processes within this demographic.

Attitude toward the behavior encompass a range of beliefs regarding the likelihood of outcomes associated with specific behaviors (54). Generally, a more favorable attitude towards a behavior correlates with a stronger intention to engage in that behavior, thereby increasing the likelihood of its actual occurrence. Research by Fazio and Zanna (73) indicates that individuals often form positive or negative expectations about behavioral outcomes based on direct experiences, with attitudes grounded in such experiences exerting a greater influence on behavior than those stemming from indirect experiences. In previous vaccination campaigns, including those for the H7N9 virus, hepatitis B, and COVID-19, there were no significant gender differences observed in the information environments fostered on social media platforms. Attitudes towards these vaccines primarily derived from the accumulation of direct past experiences, which were not markedly affected by sex-biased information. In contrast, the current dynamics surrounding HPV vaccination reveal distinct characteristics: attitudes towards HPV vaccination are considerably shaped by a sex-biased information environment, diverging from the mechanisms influencing other vaccines. Consequently, sex bias has not been recognized as a primary factor in previous investigations into vaccine attitudes and vaccination behaviors (74–77).

Within the Chinese context, HPV vaccines are classified into two categories: one that is provided at no cost under the national immunization program and another that requires out-of-pocket payment. The disparity in vaccine pricing significantly influences individuals’ attitudes towards vaccination and their actual vaccination behaviors. For instance, research conducted in Asian nations has shown that public willingness to vaccinate often declines markedly when confronted with higher-priced private vaccines (78). In summary, investigations into HPV vaccine uptake among women indicate that attitudes toward the vaccine can effectively predict vaccination behavior, with more positive attitudes leading to a greater willingness to vaccinate, although this relationship is moderated by the vaccine’s price (38, 79–81). Therefore, a positive attitude towards HPV vaccination should also be regarded as a critical factor in forecasting HPV vaccination rates among males in China. Therefore, this study proposes:

*H1: Attitudes toward HPV vaccination behavior positively influence (a) the willingness of males to pay for HPV vaccination and (b) the willingness to receive free HPV vaccination.*

Subjective Norms are defined as an individual’s perception of societal expectations that either endorse or discourage a particular behavior (54). Cultural contexts significantly shape health perceptions, resulting in varying interpretations and narratives regarding the same illness in different countries, regions, and even within the same nation. From a cultural perspective, China is a collectivist society, where social values are centered on collective interests. In collectivist societies, when making decisions about specific behaviors, individuals place greater emphasis on the support and opposition of others and groups in society, and are more inclined to conform to social expectations (82). In China, the vaccination behaviors of individuals are intricately associated with their sense of social responsibility. Studies suggest that individuals who possess a heightened sense of social responsibility are more likely to engage in vaccination, particularly evident during the COVID-19 vaccination campaign (83, 84). Furthermore, existing research indicates that subjective norms play a crucial role in influencing HPV vaccination rates among women (27, 35, 38, 66). Therefore, it is reasonable to propose that subjective norms, including the concept of social responsibility, also impact HPV vaccination behaviors among male populations in China. Therefore, the present study posits:

*H2: The subjective norms regarding HPV vaccination have a positive influence on (a) the willingness of male populations to pay for HPV vaccination and (b) their willingness to receive free HPV vaccination.*

Perceived behavioral control refers to an individual’s belief in their ability to manage and perform a specific behavior, regardless of its complexity (54). Economic capability stands as a significant determinant for individuals when evaluating their decision-making capacities. In research targeting female populations, economic factors—including family income and socioeconomic status—have been found to have a strong correlation with HPV vaccination rates (85, 86). Most of these studies indicate that perceived behavioral control serves as a positive predictor of HPV vaccination among women (23, 38, 66). Therefore, it is plausible to assert that perceived behavioral control regarding HPV vaccination among Chinese men will similarly serve as a positive predictor of their intention to receive the HPV vaccine (66, 70, 87). Therefore, we propose:

*H3: Perceived behavioral control of HPV vaccination has a positive impact on (a) the willingness of men to pay for HPV vaccination and (b) the willingness of men to receive free HPV vaccination.*

This research also examines the influence of sex-biased information on the willingness of male populations to receive the HPV vaccination. Consequently, drawing from prior research and the theoretical framework outlined earlier, sex-biased information is introduced as a precursor variable within the TPB model, thereby developing an extended TPB model that incorporates sex-biased information. Thus, the current study aims to explore the following research questions:

*RQ1: Does sex-biased HPV vaccination information have a direct impact on the HPV vaccination intentions of male individuals?*

*RQ2: Does sex-biased HPV vaccination information exert an indirect influence on the HPV vaccination intentions of male individuals through (a) attitudes toward HPV vaccination behavior, (b) subjective norms regarding HPV vaccination, and (c) perceived behavioral control over HPV vaccination?*

**Figure 1:**
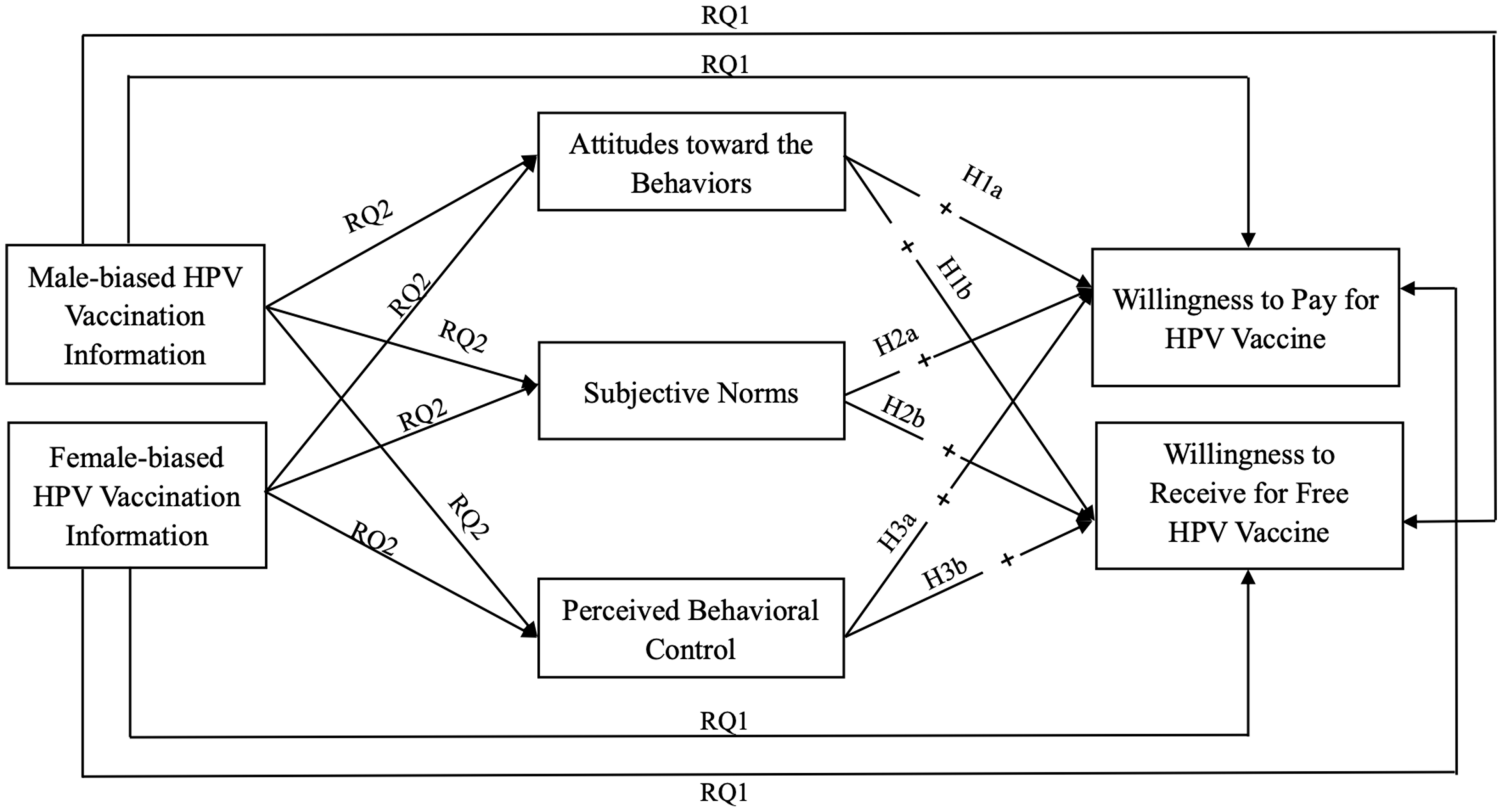
Theoretical framework.

Overall, this study, which adopts a male viewpoint and employs TPB, develops an enhanced TPB model that incorporates the concept of information sex bias. The research investigates the impact of sex-biased information on the intention of men to receive the HPV vaccination, while also examining the mechanisms that underlie this relationship. The proposed extended TPB model, informed by sex-biased information, is illustrated in Figure 1.

## 3 Methods

### 3.1 Data and sample

The methodology employed in this study was a questionnaire survey. The questionnaires were disseminated via the WJX.cn platform on Chinese social media channels. Our team distributed questionnaires online in mainland China during September to October 2024. Participants provided informed consent and received a random reward ranging from RMB 0.5 to 3 upon completing the questionnaire. Given that all items in the questionnaire were mandatory, there were no missing values in the data. However, we established criteria for identifying and removing outliers. First, attention check questions were included in the survey; responses indicating “no” to the item stating “Please select ‘yes’“were excluded from the dataset. Second, responses that were completed in less than 100 s were also removed from analysis.

Given that the peak age for male patients positive for HPV across various provincial regions in China is between 20 and 39 years (88), which coincides with the age demographic of students in higher education institutions, the participants in this research were male students from colleges and universities. A convenience sampling approach was utilized to survey individuals aged 18 years and older within this target group. Regarding the sample size of this study, as we ultimately construct a structural equation model for statistical analysis, the widely accepted and cited assertion that “at least 100–200” samples are required is supported by references such as Principles and Practice of Structural Equation Modeling [(89), p. 16]. The sample size should be at least 5–10 times the maximum path length in the model. MacCallum et al. (90) extensively discussed various factors influencing SEM sample size and proposed the 5–10 times guideline as a general principle. In our model, the longest path comprises three variables, indicating a required sample size of approximately 150 to 300 participants. Accordingly, we set our target at the midpoint of this range, which is 225. After collecting a total of 264 questionnaires and discarding invalid responses, we achieved a final valid sample size of 240, thereby meeting the established criteria, also resulting in an effective recovery rate of 90.23%. However, it is important to note that due to constraints associated with the research topic, many potential respondents were reluctant to engage in surveys concerning male HPV issues. This limitation may regrettably hinder our ability to secure a larger sample size.

The sample comprised individuals aged between 19 and 30 years (*M* = 22.2, *SD* = 1.98), and included 16 junior college students (6.7%), 187 undergraduate students (77.9%), 36 master’s students (15.0%), and 1 doctoral student (0.4%). Regarding sexual orientation, 220 participants identified as heterosexual (91.7%), while 20 participants identified as homosexual, bisexual, or other orientations (8.3%). The findings indicated that 107 male college students (44.6%) reported having prior sexual experience, of whom 17 (15.9% of those with sexual experience) indicated having engaged in sexual relations with individuals of the same sex. Furthermore, a total of 65 male college students (27.1%) within the sample had received the HPV vaccination (see Table 1).

**Table 1:** Sample characteristics (*N* = 240).

Variable	*M* (*SD*) or *N* (%)
Demographic characteristics	
Age	22.2 (1.98)
Education level	
Junior college	16 (6.7%)
Bachelor degree	187 (77.9%)
University master	36 (15.0%)
University doctor	1 (0.4%)
Sexual orientation	
Heterosexuality	220 (91.7%)
Homosexual/Bisexual/Other	20 (8.3%)
Self-reported sexual behavior	
Sexual history	
Yes	107 (44.6%)
No	133 (55.4%)
Homosexual history	
Yes	17 (15.9%)
No	90 (84.1%)
HPV vaccination history	
Yes	65 (27.1%)
No	175 (72.9%)

### 3.2 Measurement

#### 3.2.1 Demographic variables

Demographic factors included age, education level (0 = college diploma, 1 = bachelor’s degree, 2 = master’s degree, 3 = doctoral degree), sexual orientation (0 = heterosexual, 1 = homosexual/bisexual/other), self-reported sexual behavior such as sexual history (0 = yes, 1 = no), homosexual history (0 = yes, 1 = no), and HPV vaccination history (0 = yes, 1 = no).

#### 3.2.2 Sex-biased HPV vaccination information

Sex-biased HPV vaccination information was measured as male-biased HPV vaccination information (HPV-MI) and female-biased HPV vaccination information (HPV-WI). The questionnaire was adapted based on prior research (91). The dimensions assessed included self-publishing, friend publishing, and media publishing. Self-published information is generally rooted in individual experiences, opinions, and emotions, and is shaped by external inputs from friends and media. Thus, self-publishing was integrated into the analysis. Conversely, media-published information is predominantly factual, data-driven, and centered on public events, encompassing dimensions related to social phenomena, public affairs, and cultural dynamics. This type of information is typically more objective, reflecting societal norms and shared characteristics. The frequency of information shared by friends falls between these two extremes. Both variables are measured using three items (1 = I never, 2 = Once a month, 3 = Every few weeks; 4 = Once a week; 5 = Every day), including: (1) “How frequently have you posted or mentioned information related to HPV vaccination for males/females on social media platforms in the past 6 months”; (2) “How frequently have your friends posted or mentioned information related to HPV vaccination for males/females on social media platforms in the past 6 months”; (3) “How frequently have you seen information related to HPV vaccination for males/females on social media platforms in the past 6 months” (where “Male-biased HPV vaccination information” items: *M* = 1.731, *SD* = 0.062, Cronbach’s *α* = 0.852; “Female-biased HPV vaccination information” items: *M* = 2.022, *SD* = 0.112, Cronbach’s α = 0.744).

#### 3.2.3 Attitudes towards HPV vaccination behaviors

The concept of attitudes towards HPV vaccination behaviors (HPV-AB) encompasses individuals’ subjective assessments pertaining to human papillomavirus (HPV) vaccination, including the preventive effect of HPV vaccine, its maintenance effect, the gains and losses of not getting vaccinated, and the benefits of vaccination. The questionnaire employs a Likert 5-point scale (1 = strongly disagree, 5 = strongly agree) and is borrowed from previous research (92). There are four items: (1) “Getting the HPV vaccine can prevent human papillomavirus infection”; (2) “The effect of getting the HPV vaccine is good”; (3) “Not getting the HPV vaccine makes it easier to be infected with human papillomavirus”; (4) “Getting the HPV vaccine can reduce the expenses due to human papillomavirus infection” (*M* = 4.01, *SD* = 0.614, Cronbach’s *α* = 0.704).

#### 3.2.4 HPV vaccination subjective norms

The HPV Vaccination Subjective Norm (HPV-SN) is a construct that assesses the perceived social pressure individuals experience when considering the HPV vaccination. This social pressure may arise from various sources, including family members, friends, governmental entities, and media representations. The measurement of HPV-SN consists of five specific items and utilizes a 5-point Likert scale (1 = strongly disagree, 5 = strongly agree) (92): (1) “My relatives and friends support me getting the HPV vaccine”; (2) “I would get the HPV vaccine because my relatives and friends recommend it”; (3) “I would get the HPV vaccine because the government provides adequate HPV vaccines”; (4) “I would get the HPV vaccine because the government provides free HPV vaccines”; (5) “I get the HPV vaccine because it is promoted for use” (*M* = 3.88, *SD* = 0.931, Cronbach’s *α* = 0.832).

#### 3.2.5 Perceived behavioral control of HPV vaccination

The Perceived Behavioral Control of HPV Vaccination (HPV-PBC) assesses individuals’ perceived ease of self-administration of the HPV vaccine. This construct encompasses several factors, including the complexity of personal decision-making, the availability of time for vaccination, the accessibility of vaccination services, and financial considerations associated with vaccination. The measure consists of four statements in a Likert scale (92) (1 = strongly disagree, 5 = strongly agree): (1) “I can decide for myself whether to get the HPV vaccine”; (2) “I have time to get the HPV vaccine”; (3) “It is convenient to get the HPV vaccine”; (4) “The cost of getting the HPV vaccine is not significant” (*M* = 3.8, *SD* = 0.920, Cronbach’s *α* = 0.704).

#### 3.2.6 Willingness to HPV vaccination

The dependent variable was defined by participants’ self-reported willingness to receive the HPV vaccination either at their own expense (HPV-P) or at no cost (HPV-F). The item was “I am willing to (pay for/receive for free) the HPV vaccine” (*M_pay_* = 3.34, *SD_pay_* = 1.003; *M_free_* = 4.35, *SD_free_* = 0.620).

All variables have been restructured based on previously established scales. A detailed account of the constructs, including Sex-biased HPV Vaccination Information, Attitudes Towards HPV Vaccination Behaviors, Subjective Norms Regarding HPV Vaccination, Perceived Behavioral Control over HPV Vaccination, and Willingness to Receive HPV Vaccination, is presented in Table 2.

**Table 2:** Details of questionnaire adaptation.

Previous research inventory	This study adapted the scale
Sex-biased HPV vaccination information	
In the past 6 months, how often did you post or mention e-cigarettes on (insert platform)?	How frequently have you posted or mentioned information related to HPV vaccination for males/females on social media platforms in the past 6 months?
In the past 6 months, how often did a friend post or mention e-cigarettes on (insert platform)?	How frequently have your friends posted or mentioned information related to HPV vaccination for males/females on social media platforms in the past 6 months?
In the past 6 months, how often did you see advertisements about e-cigs on (insert platform)?	How frequently have you seen information related to HPV vaccination for males/females on social media platforms in the past 6 months?
Attitudes towards HPV vaccination behaviors	
Getting the EV71 vaccine can prevent Hand, foot and mouth disease.	Getting the HPV vaccine can prevent human papillomavirus infection.
The effect of getting the EV71 vaccine is good.	The effect of getting the HPV vaccine is good.
Not getting the EV71 vaccine makes it easier to be infected with Hand, foot and mouth disease.	Not getting the HPV vaccine makes it easier to be infected with human papillomavirus.
Getting the EV71 vaccine can reduce the expenses due to Hand, foot and mouth disease.	Getting the HPV vaccine can reduce the expenses due to human papillomavirus infection.
HPV vaccination subjective norms	
My relatives and friends support me getting the EV71 vaccine for my children.	My relatives and friends support me getting the HPV vaccine.
I would get the EV71 vaccine for my children because my relatives and friends recommend it	I would get the HPV vaccine because my relatives and friends recommend it.
I would get the EV71 vaccine for my children because the government provides HPV vaccines.	I would get the HPV vaccine because the government provides adequate HPV vaccines.
	I would get the HPV vaccine because the government provides free HPV vaccines.
I get the EV71 vaccine for my children because it is promoted for use.	I get the HPV vaccine because it is promoted for use.
Perceived behavioral control of HPV vaccination	
I can decide for my children whether to get the EV71 vaccine.	I can decide for myself whether to get the HPV vaccine.
I have time to get the EV71 vaccine for my children.	I have time to get the HPV vaccine.
It is convenient to get the EV71 vaccine for my children.	It is convenient to get the HPV vaccine.
The cost of getting the EV71 vaccine is not significant.	The cost of getting the HPV vaccine is not significant.
Willingness to HPV vaccination	
I am willing to pay for the EV71 vaccine for my children.	I am willing to pay for the HPV vaccine.
I am willing to get the EV71 vaccine for my children.	I am willing to receive for free the HPV vaccine.

### 3.3 Data analysis

This research employed SPSS 26.0 and Amos 28.0 software for the analysis of data results. Initially, SPSS 26.0 was used to evaluate the reliability and validity of the questionnaire. The findings from the reliability assessment indicated that the Cronbach’s alpha coefficients for each primary variable ranged from 0.704 to 0.852, exceeding the threshold of 0.7, which suggests that the questionnaire demonstrates adequate reliability. Furthermore, the examination of structural validity revealed the following: (1) The KMO statistic and Bartlett’s test of sphericity for each variable yielded a KMO value of 0.808, which is above the 0.7 threshold, indicating that the intercorrelations among the variables are not significantly different, thus rendering the data highly appropriate for factor analysis (93); (2) The *p*-values obtained from Bartlett’s test of sphericity were consistently 0.000, which is below the 0.001 level, leading to the rejection of the sphericity hypothesis and confirming the presence of correlations among the original variables, thereby affirming the suitability of the data for factor analysis.

Subsequently, a confirmatory factor analysis was performed utilizing AMOS 28.0 software to assess a measurement model comprising five latent variables: HPV-MI (HPV vaccination information favoring males), HPV-WI (HPV vaccination information favoring females), HPV-AB (behavioral attitudes toward HPV vaccination), HPV-SN (subjective norms pertaining to HPV vaccination), HPV-PBC (perceived behavioral control over HPV vaccination), as well as two observed variables, HPV-P (self-funded HPV vaccination) and HPV-F (complimentary HPV vaccination). The evaluation metrics employed were convergent validity and discriminant validity. The findings demonstrated that the overall fit indices of the measurement model were satisfactory, with the following values recorded: χ^2^ = 353.196, χ^2^/df = 2.102, RMSEA = 0.068, CFI = 0.909, and IFI = 0.911. The composite reliability (CR) values exceeded 0.7, and the average variance extracted (AVE) values were all greater than 0.35, confirming the high convergent validity of the measurement scale (see Table 3).

**Table 3:** Potential dimension reliability analysis.

Dimension	Latent variable	Standardized regression weights	Regression weights	*S.E.*	T-value	*P*	SMC	CR	AVE
HPV-MI	HPV-MI_3	0.745	1.000				0.555	0.866	0.685
	HPV-MI_2	0.890	1.061	0.077	13.792	***	0.792		
	HPV-MI_1	0.841	0.832	0.060	13.848	***	0.707		
HPV-AB	HPV-AB_4	0.712	1.000				0.507	0.737	0.417
	HPV-AB_3	0.467	0.826	0.131	6.294	***	0.218		
	HPV-AB_2	0.678	0.802	0.095	8.479	***	0.460		
	HPV-AB_1	0.696	0.739	0.085	8.660	***	0.484		
HPV-SN	HPV-SN_5	0.746	1.000				0.557	0.838	0.510
	HPV-SN_4	0.642	0.794	0.085	9.377	***	0.412		
	HPV-SN_3	0.770	1.059	0.091	11.698	***	0.593		
	HPV-SN_2	0.759	0.978	0.088	11.139	***	0.576		
	HPV-SN_1	0.641	0.847	0.092	9.228	***	0.411		
HPV-PBC	HPV-PBC_4	0.414	1.000				0.171	0.689	0.365
	HPV-PBC_3	0.595	1.334	0.219	6.102	***	0.354		
	HPV-PBC_2	0.736	1.468	0.287	5.115	***	0.542		
	HPV-PBC_1	0.628	1.184	0.248	4.778	***	0.394		
HPV-WI	HPV-WI_3	0.619	1.000				0.383	0.784	0.557
	HPV-WI_2	0.939	1.395	0.154	9.037	***	0.882		
	HPV-WI_1	0.637	0.905	0.101	8.945	***	0.406		

^*^p < 0.05, ^**^p < 0.01, ^***^p < 0.001.

Before implementing the specific verification model, we conducted a correlation analysis among the various variables, with detailed results presented in Table 4. Additionally, we performed a multicollinearity assessment on the variables included in the model. The findings indicated that there were no multicollinearity issues among the variables incorporated into the model. Specifically, the minimum variance inflation factor (VIF) value was 1.267, while the maximum VIF value reached 1.778, both of which met the critical threshold of VIF < 10 (117: 102).

**Table 4:** Correlation analysis.

Variables	*M*	*SD*	1	2	3	4	5	6
1 HPV-AB	15.940	2.666						
2 HPV-SN	19.323	3.677	0.537^**^					
3 HPV-PBC	15.149	2.854	0.433^**^	0.575^**^				
4 HPV-MI	12.746	2.435	−0.077	−0.129^*^	−0.187^**^			
5 HPV-WI	11.907	2.402	−0.050	−0.091	−0.005	0.442^**^		
6 HPV-P	3.330	1.000	0.278^**^	0.532^**^	0.516^**^	−0.279^**^	−0.082	
7 HPV-F	4.320	0.810	0.509^**^	0.646^**^	0.515^**^	−0.047	−0.082	0.326^**^

^*^p < 0.05, ^**^p < 0.01.

## 4 Results

The results of the path analysis for the extended TPB model were derived using structural equation modeling, with the corresponding path coefficients presented in Table 5.

**Table 5:** TPB model path coefficient.

Pathway	Estimate	*P*	95% CI		*S.E.*	C.R.
			Lower	Upper		
HPV-MI→HPV-P	0.215^***^	***	0.160	0.374	0.089	3.192
HPV-WI→HPV-P	0.029	0.657	−0.005	0.211	0.100	0.444
HPV-MI→HPV-F	−0.098	0.060	−0.018	0.155	0.063	−1.622
HPV-WI→HPV-F	0.057	0.328	−0.043	0.150	0.072	0.978
HPV-MI→HPV-AB	0.187^*^	0.040	0.043	0.199	0.076	2.057
HPV-WI→HPV-AB	0.096	0.280	−0.022	0.167	0.087	1.080
HPV-AB→HPV-P	−0.182	0.060	−0.074	0.323	0.152	−1.884
HPV-AB→HPV-F	0.208^*^	0.015	0.185	0.435	0.106	2.436
HPV-MI→HPV-SN	0.215^*^	0.011	0.043	0.210	0.079	2.549
HPV-WI→HPV-SN	0.044	0.600	−0.022	0.160	0.092	0.525
HPV-SN→HPV-P	0.429^***^	***	0.260	0.536	0.156	3.891
HPV-SN→HPV-F	0.312^**^	0.002	0.243	0.517	0.109	3.174
HPV-MI→HPV-PBC	0.247^*^	0.025	0.002	0.214	0.065	2.235
HPV-WI→HPV-PBC	−0.043	0.634	−0.041	0.082	0.063	−0.477
HPV-PBC→HPV-P	0.265^*^	0.017	0.089	0.416	0.249	2.388
HPV-PBC→HPV-F	0.327^**^	0.005	0.124	0.345	0.204	2.835

^*^p < 0.05, ^**^p < 0.01, ^***^p < 0.001.

Information regarding HPV vaccination that is biased towards males has a substantial impact on various factors related to the willingness to pay for the HPV vaccine. Specifically, the analysis reveals that such information significantly affects self-reported willingness to pay for vaccination (*β* = 0.215, *SE* = 0.089, *p* < 0.001), attitudes towards HPV vaccination behaviors (β = 0.187, *SE* = 0.076, *p* = 0.040), subjective norms associated with HPV vaccination (β = 0.215, *SE* = 0.079, *p* = 0.011), and perceived behavioral control over HPV vaccination (β = 0.247, *SE* = 0.065, *p* = 0.025). Overall, the findings indicate that male-biased HPV vaccination information exerts a positive influence on individuals’ behavioral attitudes, subjective norms, perceived control, and their willingness to invest in vaccination.

The analysis revealed no significant differences in the willingness to pay for or accept free HPV vaccinations, as well as in attitudes towards HPV vaccination behavior, subjective norms, and perceived behavioral control among individuals exposed to sex-biased HPV vaccination information. This finding suggests that such information exerts neither a positive nor a negative influence on the aforementioned variables. Therefore, RQ1 and RQ2 have been addressed.

A significant difference was identified in the willingness to accept free HPV vaccination, contingent upon individuals’ attitudes towards HPV vaccination behaviors (*β* = 0.208, *SE* = 0.106, *p* = 0.015). This finding suggests that positive attitudes towards HPV vaccination behaviors significantly enhance the likelihood of accepting free HPV vaccination. Therefore, H1b is supported, while H1a is not.

The subjective norms associated with HPV vaccination had a significant impact on individuals’ willingness to pay for the vaccine (*β* = 0.429, *SE* = 0.156, *p* < 0.001) as well as their willingness to accept a free HPV vaccination (β = 0.312, *SE* = 0.109, *p* = 0.002). These findings indicate that subjective norms exert a positive influence on the willingness to either pay for or receive a complimentary HPV vaccination. Therefore, H2a and H2b are supported.

**Figure 2:**
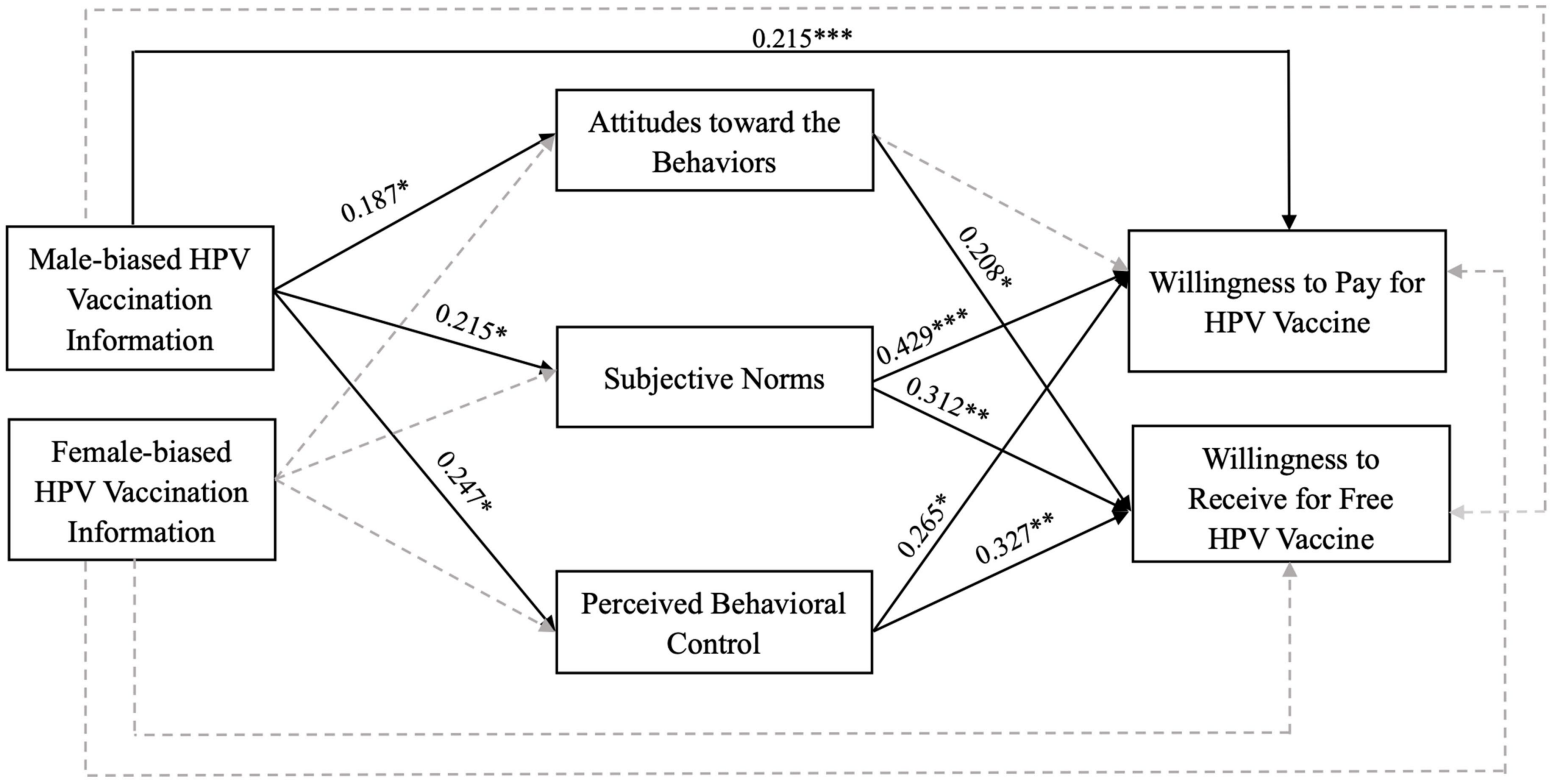
Model results.

The perceived behavioral control regarding HPV vaccination plays a significant role in influencing individuals’ willingness to pay for the vaccine (β = 0.265, *SE* = 0.249, *p* = 0.017) as well as their willingness to accept free HPV vaccination (β = 0.327, *SE* = 0.204, *p* = 0.005). These findings suggest that individuals who perceive a greater degree of control over HPV vaccination are more likely to express a willingness to both pay for and receive the vaccination at no cost. Therefore, H3a and H3b are supported. The overall framework of the extended TPB model, which incorporates considerations of gender bias, is presented in Figure 2.

In summary, sex-biased information regarding HPV vaccination can have both direct and indirect effects on the vaccination behaviors of male college students. More specifically, male-biased information regarding HPV vaccination is associated with an enhancement in attitudes toward vaccination behavior, subjective norms, and perceived behavioral control. This, in turn, directly influences the willingness to pay for HPV vaccination and indirectly impacts both the willingness to pay and the willingness to accept free HPV vaccination. Conversely, female-biased information does not exert any significant positive or negative influence on these variables.

## 5 Discussion

Existing research on the determinants of HPV vaccination behavior predominantly concentrates on female populations. This study expands upon this foundation by examining the effects of sex-biased information on HPV vaccination behaviors among male populations. The results indicate that sex-biased information can exert both direct and indirect influences on the health behaviors of male individuals. This paper posits the following conclusions: (1) the relationship between such information and an individual’s gender identity is notable; (2) sex-biased information activates distinct gender concepts, thereby prompting individuals to employ varying methods of information processing; (3) the financial cost of the vaccine emerges as a significant factor contributing to hesitancy toward HPV vaccination.

### 5.1 Promotion of vaccination intentions through a male-biased information environment

The current research indicates that targeted information positively influences male college students’ attitudes, subjective norms, and perceived control concerning HPV vaccination, ultimately enhancing their willingness to either pay for or accept free vaccination. When male college students are exposed to information specifically addressing HPV vaccination for males, they receive affirmative messages regarding the vaccine’s safety, efficacy, and its role in preventing diseases associated with persistent HPV infection. Such exposure is likely to foster more favorable attitudes towards vaccination, thus increasing the likelihood of participation. Furthermore, the prevalence of HPV-related information aimed at males, once it reaches a critical threshold within the information environment, can generate social pressure among male college students. This pressure arises from a societal consensus that views HPV vaccination as a responsible action. Recommendations from family members, friends, and healthcare professionals can further amplify this social pressure, making individuals more inclined to pursue vaccination.

Perceived behavioral control, which is shaped by perceptions of difficulty and control (118), plays a crucial role in predicting behavioral intentions. Notably, perceived difficulty tends to exert a stronger influence than perceived control, and it is significantly affected by key message components. When male individuals are exposed to essential information—such as guidelines on how and when to receive the HPV vaccine—they experience a reduction in perceived difficulty, leading to an enhancement in perceived behavioral control (94). This, in turn, influences their behavioral intentions. Thus, the dissemination of core informational elements, whether through publications, discussions with peers, or social media platforms, captures male attention and stimulates cognitive engagement. This cognitive engagement diminishes the perceived difficulty associated with obtaining the HPV vaccine, prompting males to modify or refine their vaccination strategies in response to new information or circumstances. As a result, HPV vaccine information specifically designed for males can more effectively reshape their perceptions and assessments regarding vaccination, significantly increasing their willingness to receive the HPV vaccine, whether at their own expense or at no cost. To improve the rate of HPV vaccination among males, it is essential to implement an information dissemination strategy that is specifically tailored to this demographic.

### 5.2 Challenges within a female-biased information environment

In our constructed model, certain pathways exhibit statistical insignificance. The primary group of non-significant pathways relates to the direct and indirect impacts of female-biased information on men’s willingness to receive the HPV vaccine, whether provided through free vaccination programs or paid services. We suggest that entrenched gender roles may contribute to this phenomenon. Men may perceive HPV risks as predominantly associated with females, thus developing a psychological defense mechanism that renders them less receptive to health promotion messages (95). Furthermore, the framing of information appears insufficient; existing female-biased narratives surrounding the HPV vaccine, particularly in advertisements, tend to emphasize cervical cancer prevention while neglecting male-specific risks such as anal and oropharyngeal cancers. This oversight fails to address critical health concerns pertinent to men (96), ultimately hindering the ability of female-biased information to influence men’s willingness to receive the HPV vaccine, irrespective of cost considerations.

From a cognitive theoretical perspective, we propose a potential explanation for this phenomenon. According to (119) Gender Schema Theory, men may often process information through the lens of socially constructed gender schemas, which leads them to prioritize information that aligns with traditional masculine roles—such as competition and goal-oriented behavior—while overlooking or minimizing information associated with femininity, such as emotional support and caregiving. This cognitive framework influences how attention is allocated. Consequently, when men encounter information regarding HPV vaccination, they tend to categorize it based on gender labels. Through these socially developed gender schemas, men may automatically classify ‘HPV vaccination’ as relevant primarily to the ‘female domain.’ When presented with the term ‘female HPV,’ men may activate cognitive filtering mechanisms due to a perceived incongruence with their gender role expectations, resulting in either neglect or minimization of the information. Conversely, if HPV vaccines are framed as something that “men can also receive,” this reframing can integrate the topic into acceptable male gender schemas by removing the “feminine” label—such as highlighting that men are also at risk for contracting HPV—thereby attracting their attention. Several studies examining men’s cognition and attitudes towards HPV and its vaccine have identified low vaccination rates among men as being linked to misunderstandings about HPV and a belief that it is not pertinent to them (97–99). This may lead to a negligible effect on health education regarding HPV among male university students.

Additionally, several mediating variables have not demonstrated their mediating effects under the influence of female-biased information. On one hand, such information lacks content specifically targeting male audiences, which diminishes its potential to foster positive perceptions regarding the HPV vaccine among men (100). On the other hand, there is currently a deficit of male opinion leaders advocating for HPV vaccination (101). The absence of gender-specific service guidance in female-biased messaging—such as designated appointment channels for male vaccinations—further inhibits the activation of perceived behavioral control (102).

The non-significant relationship observed in the second group indicates that male-biased information regarding HPV vaccination does not have a meaningful direct impact on the willingness to accept free HPV vaccination. This lack of a direct effect may be explained by the cost sensitivity paradox. Researchers suggest that when a service is offered for free, it can lead to a “zero-price effect,” whereby individuals believe that free products decrease their production costs (103). Considering this phenomenon, the current study posits that the provision of free HPV vaccinations could unintentionally heighten men’s doubts about the quality of the vaccine. Unlike information targeted at females, which may encounter less skepticism due to the availability of free HPV vaccination programs for women in certain regions of China, men are likely to prioritize vaccine quality and question the effectiveness of complimentary vaccines. Consequently, their willingness to pay for vaccination can be shaped by subjective norms—such as messages from parents, friends, or healthcare professionals promoting male HPV vaccination (104)—and perceived behavioral control, which includes guidance from healthcare providers regarding the practicality and benefits of receiving vaccinations (105).

Similarly, the lack of a significant path in the third group suggests that people’s attitudes towards vaccination do not significantly affect their willingness to pay for it. This is mainly due to economic considerations that diminish the impact of positive attitudes. Studies have shown that the costs associated with vaccines can reduce the positive feelings people have about vaccination and act as a major obstacle to getting vaccinated (13, 106–108). As a result, individuals’ attitudes cannot reliably predict men’s willingness to pay for vaccinations. We will also explore the mechanisms through which price influence vaccination behavior in subsequent discussions.

### 5.3 Understanding the psychological logic of male HPV vaccination behavior through effect size

In accordance with the effect size criteria established by Kline (89), where a *β*-value of 0.10 signifies a small effect, 0.30 indicates a medium effect, and 0.50 represents a large effect, the independent variable “male-biased HPV vaccination information” demonstrates notable effects on three variables outlined in the TPB: attitudes toward the behavior (β = 0.187), subjective norms (β = 0.215), and perceived behavioral control (β = 0.247). All these effects are classified as small, with perceived behavioral control approaching the threshold for a medium effect. This finding implies that gender-biased information is particularly effective in influencing perceived behavioral control. According to the theoretical framework of Ajzen (54), perceived behavioral control refers to an individual’s personal assessment of their capability to successfully engage in a specific behavior, which encompasses their understanding of potential challenges they may face during its execution. Male-biased information typically manifests in the form of prescriptive texts that instruct men on when, how, and where to receive vaccinations. Such information plays a vital role in shaping individuals’ perceptions regarding the practical challenges associated with HPV vaccination.

The effects of the variables from TPB on individuals’ willingness to vaccinate differ across various contexts. In the scenario of free vaccination, both subjective norms (*β* = 0.312) and perceived behavioral control (β = 0.327) exhibit moderate effect sizes, whereas attitudes toward vaccination behaviors (β = 0.208) are characterized by a small effect size. This suggests that the inclination to accept free vaccination is more significantly influenced by the interaction between social norms and individuals’ self-assessed capabilities, highlighting the importance of personal beliefs regarding the necessity and method of receiving the HPV vaccine. Conversely, in scenarios involving paid vaccination, subjective norms (*β* = 0.429) exert a notably stronger influence compared to their impact on willingness for free vaccination. This implies that when vaccination incurs a cost, individuals’ decisions are largely guided by the preferences expressed by significant others concerning their vaccination status. The effect of perceived behavioral control in this context is relatively small (*β* = 0.265), which may indicate that financial considerations act as a considerable impediment to engaging in vaccination behavior. For men specifically, concerns regarding economic barriers may diminish their perception of control over these barriers, leading to a lesser impact overall. Furthermore, the direct effect of “male-targeted HPV vaccination information” on “willingness to pay for HPV vaccine” is also classified as having a small effect size (β = 0.215). This underscores that in order to enhance outcomes related to vaccination willingness and behavior, it may be advantageous to leverage TPB mediating pathways for a more comprehensive influence.

### 5.4 The mechanism of Price influence on HPV vaccine uptake among males

The economic implications associated with HPV vaccines may negatively affect the attitudes of male college students towards vaccination. Our research demonstrates that favorable attitudes towards vaccination among male college students enhance their willingness to accept free HPV vaccinations; however, no significant difference is observed in their propensity to pay for these vaccinations. Additionally, subjective norms have a positive effect on both their willingness to pay for and to receive free HPV vaccinations, while perceived behavioral control also positively influences both aspects of vaccination willingness. Information regarding HPV vaccination targeted at men predominantly emphasizes the vaccine’s protective benefits against HPV, which can foster positive perceptions and attitudes towards vaccination. Nevertheless, in scenarios where individuals must finance their own vaccinations, economic costs emerge as a critical variable influencing decision-making, thereby diminishing the role of attitudes in vaccination choices. Consequently, attitudes towards vaccination exert varying levels of influence depending on the economic context.

In the framework of the Theory of Planned Behavior, economic factors primarily shape behavioral attitudes, rather than subjective norms or perceived behavioral control. This differentiation arises from the theory’s classification of distinct psychological constructs, each of which corresponds to unique psychological processes and social influences. Behavioral attitudes inherently encompass considerations of economic factors, with economic costs forming a substantial component of overall behavioral costs. Conversely, subjective norms focus on the social pressures individuals encounter, while perceived behavioral control pertains to individuals’ internal assessments of their capabilities. The latter two constructs involve behavioral costs that are largely unassociated with economic factors. Perceived behavioral control and subjective norms have a significant impact on intentions to receive both free and paid HPV vaccinations. Subjective norms pertain to individuals’ perceptions of social expectations from family, friends, and society regarding male HPV vaccination. On the other hand, perceived behavioral control relates to beliefs about one’s capability to carry out the vaccination behavior, including factors like access to vaccination services, affordability, and logistical considerations. These elements emerged as important predictors of vaccination intentions. Regardless of vaccination cost, male college students integrated social norms (i.e., what others expect of them) and self-assessed capabilities (i.e., whether they could practically obtain the vaccine) into their decision-making. This aligns with theoretical distinctions between subjective norms (social pressure to conform) and perceived behavioral control (perceived ease or difficulty of action), highlighting their unique roles in shaping health behaviors (120).

Information regarding HPV vaccination that is specifically targeted towards males appears to mitigate price sensitivity towards HPV vaccines by amplifying the perceived threat of HPV. Such informational cues play a significant role in shaping individuals’ perceptions of disease threats, encompassing both susceptibility and severity, thereby promoting proactive health behaviors (109). The findings of this study suggest that male-targeted HPV vaccination information positively influences men’s willingness to pay for HPV vaccines, while there is no notable difference in their willingness to accept free HPV vaccines. Researchers contend that the pricing of vaccines is a crucial determinant of vaccine hesitancy among the public (68), which subsequently impacts individuals’ choices regarding the timing of health behaviors, including HPV vaccination. The informational landscape surrounding HPV vaccination for men, influenced by personal experiences, peer discussions, and social media, encompasses essential knowledge such as the appropriate age for vaccination and the procedural aspects, as well as more nuanced medical insights regarding the significance of male HPV vaccination, including prevalence, incidence, and transmission pathways. This context facilitates a shift in male college students’ perceptions of the health threats posed by HPV, bolstering their awareness of both susceptibility and severity of the virus, which in turn affects their perceptions of the cost-effectiveness of vaccination and enhances their willingness to invest in out-of-pocket vaccinations.

### 5.5 Practical implications

The findings of this study suggest that tailored information regarding HPV vaccination aimed at men is likely to increase their willingness to receive the vaccine. Therefore, it is essential to enhance the dissemination of various forms of male-oriented HPV vaccine promotion, particularly through media channels, with a focus on social media platforms. By emphasizing the male perspective, specifically highlighting men’s vulnerability to HPV-related diseases, we can facilitate a greater understanding among men regarding the highly transmissible nature of HPV, which affects individuals regardless of gender. This approach also serves to underscore the effectiveness of the HPV vaccine, thereby enhancing men’s awareness and acceptance of vaccination. Moreover, implementing a range of engaging and entertaining promotional strategies could elevate the significance of HPV awareness while mitigating perceived risks associated with vaccination. For instance, in China, health campaigns such as the “Playful Immunization Promotion” initiative (110), which utilized platforms like TikTok, have demonstrated effectiveness in capturing public interest and increasing vaccination rates. Similar initiatives could be strategically developed to specifically target male audiences.

Cost sensitivity has emerged as a significant barrier to HPV vaccination among men, as identified in this study. The introduction of economic stimuli like financial subsidies for vaccine administration could serve as one of the essential strategies for increasing vaccination uptake. For instance, several regions in China, including Guangdong and Zhejiang provinces, have enacted policies that provide free HPV vaccinations to certain age groups of women (111). This provision of free vaccines is closely associated with female identity, and the messaging surrounding “free HPV vaccinations” does not directly engage men; rather, it indirectly motivates men to consider accepting free HPV vaccinations through the influence of behavioral attitudes, subjective norms, and perceived behavioral control. The sex-biased information regarding HPV vaccination significantly shapes the health behaviors of male college students, and the underlying structural factors must not be overlooked. The implementation of free vaccination initiatives, including those for HPV, is contingent upon various factors such as local fiscal allocations, public health policies, and vaccine availability. Presently, free HPV vaccination policies primarily target specific adolescent age groups rather than a wider demographic. To enhance the willingness of males, particularly male college students, to pay for HPV vaccination, it is essential to provide them with repeated exposure to HPV vaccination information that is specifically designed for a male audience.

## 6 Conclusion and limitations

This research employed a questionnaire survey approach to explore the sex-biased information environment related to HPV vaccination, delving into whether such sex-specific information has a notable effect on male individuals’ health behavior decisions, particularly their intention to receive the HPV vaccine. The study also extended the Theory of Planned Behavior model to analyze the underlying mechanisms. The results indicate that information favoring males exerts a greater influence on the vaccination intentions of male college students compared to information favoring females. In particular, the cost of the vaccine was identified as a significant barrier for male college students seeking vaccination. Additionally, consistently delivering HPV vaccination information that is tailored towards males effectively diminishes their sensitivity to price, thereby increasing their willingness to receive the vaccine.

In recent years, marketing strategies for HPV vaccines have increasingly associated the virus predominantly with women. However, it is important to recognize that HPV infection is not inherently sex-specific. To effectively combat cervical cancer, widespread vaccination among women is vital; nevertheless, the participation of men in vaccination efforts is equally important. Given that men exhibit a significant prevalence of HPV infection, it is essential to actively promote HPV vaccination initiatives targeting male populations. To enhance men’s willingness to receive the vaccine and facilitate their participation, it is crucial to improve the dissemination of information tailored specifically for men within the predominantly female-focused HPV vaccination discourse. This approach will contribute to safeguarding the sexual health of sexually active men, including those who engage in sexual activities with other men, within China’s substantial male demographic of 720 million individuals.

This study remains in its initial phases, and further investigation will enhance the understanding of the research questions posed. Currently, the article presents several limitations. First, the dataset utilized is relatively small, leading to concerns regarding its representativeness. Also, the study employs convenience sampling, which raises concerns regarding the representativeness of the sample. Specifically, web users may not accurately reflect the broader population, potentially resulting in selection bias. Additionally, the reliance on voluntary participation often leads to the selection of participants based on their interests rather than randomization, further introducing bias into the findings. Moreover, this study is based on self-reported data from participants, which may be susceptible to recall bias and the influence of social desirability. To more accurately assess the impact of sex-biased information while minimizing subjective interference, subsequent studies could implement experimental methodologies. To improve the generalizability of the findings, future research should prioritize the expansion of both the scope and scale of data collection.

Second, the current study employs a single-item measure to assess the willingness of the sample to accept both paid and free vaccines. Although this approach has been utilized in previous research (e.g., 121), the use of a single-item assessment carries inherent risks, particularly concerning reliability issues. This methodology may consequently introduce biases into the findings of this research. Furthermore, this study did not provide illustrative examples of male-biased and female-biased HPV vaccination-related information on social media to aid participants in completing the questionnaire. This omission may have hindered their understanding of the concept of “information related to male/female HPV vaccination.” Consequently, this lack of clarity could have led to a certain degree of bias in the participants’ evaluations of the items presented.

Moreover, this study is limited to a sample exclusively composed of males, thus lacking a comparative analysis that includes different gender perspectives. To achieve a more holistic understanding of the effects of sex-biased information, future research should include both male and female participants, comparing sex-specific reactions to such information to uncover potential differential impacts. Research design employing the aforementioned approach will contribute to a deeper comprehension of the role of sex-biased information in health communication and provide a scientific foundation for developing more effective health intervention strategies. In addition, our research did not assess the perceived relative cost of the HPV vaccine. Exploring this dimension may yield additional noteworthy and significant insights.

## Data Availability

The original contributions presented in the study are included in the article/supplementary material, further inquiries can be directed to the corresponding author.

## Footnotes

^1^Data source: National Bureau of Statistics of China https://data.stats.gov.cn/easyquery.htm?cn=C01
